# Development of a Sensor Node for Remote Monitoring of Plants

**DOI:** 10.3390/s19224865

**Published:** 2019-11-08

**Authors:** Alexandro Catini, Leonardo Papale, Rosamaria Capuano, Valentina Pasqualetti, Davide Di Giuseppe, Stefano Brizzolara, Pietro Tonutti, Corrado Di Natale

**Affiliations:** 1 Department of Electronic Engineering, Tor Vergata University of Rome, Via del Politecnico 1, 00133 Rome, Italy; papaleleonardo1297@gmail.com (L.P.); capuano@ing.uniroma2.it (R.C.); Valentina.Pasqualetti@uniroma2.it (V.P.); di.giuseppe@ing.uniroma2.it (D.D.G.); dinatale@uniroma2.it (C.D.N.); 2 Sant’Anna School of Advanced Studies, Piazza Martiri della Libertà 33, 56127 Pisa, Italy; s.brizzolara.sssup@gmail.com (S.B.); pietro.tonutti@santannapisa.it (P.T.)

**Keywords:** gas sensing, WSN, plant health, recursive neural network, VOCs

## Abstract

The appraisal of stress in plants is of great relevance in agriculture and any time the transport of living plants is involved. Wireless sensor networks (WSNs) are an optimal solution to simultaneously monitor a large number of plants in a mostly automatic way. A number of sensors are readily available to monitor indicators that are likely related to stress. The most common of them include the levels of total volatile compounds and CO_2_ together with common physical parameters such as temperature, relative humidity, and illumination, which are known to affect plants’ behavior. Recent progress in microsensors and communication technologies, such as the LoRa protocol, makes it possible to design sensor nodes of high sensitivity where power consumption, transmitting distances, and costs are optimized. In this paper, the design of a WSN dedicated to plant stress monitoring is described. The nodes have been tested on European privet (*Ligustrum Jonandrum*) kept in completely different conditions in order to induce opposite level of stress. The results confirmed the relationship between the release of total Volatile Organic Compounds (VOCs) and the environmental conditions. A machine learning model based on recursive neural networks demonstrates that total VOCs can be estimated from the measure of the environmental parameters.

## 1. Introduction

The standard approach for the monitoring of crops and single plants is based on the measurement of environmental parameters such as humidity, illumination, and soil composition. More recently, taking advantage of the progress of sensors and electronic technologies, attention has been focused on measuring proper plant parameters. Furthermore, nanotechnology is beginning to enable the hybridization of technology with plants in order to incorporate additional functions into the living system [1].

Among the plant parameters assuming a great importance is the measurement of released volatile compounds. These substances are synthesized and released in the environment for a multitude of functions, among them the attraction of pollinators and the repulsion of parasites [2]. Additionally, as demonstrated by several studies on humans [3], changes in the profile of VOCs can signal the occurrence of pathologies.

The displacement of observations from the environment to the single plant is a great advance because it can take into account the intrinsic individual variability of plants’ responses to environmental conditions. Furthermore, it allows adjustments to the microenvironment around each plant in order to establish the optimal farming conditions.

The health of plants can be jeopardized by exposure to either abiotic or biotic sources [4]. The most relevant abiotic factors are deficiencies or excesses of temperature, light, availability of water and concentration of nutrients in the soil, but they can also be mechanical deformations or the presence of pollutants. The biotic factors include pathogens and animals. Sensor technologies have been made available for each of these factors. However, not all of them can be applied to individual plants. For instance, biosensors have been developed for pathogen detection [5], but the technology of biosensors is still not mature enough for large-scale and long-term use of these devices. However, a large number of low-cost and high-performance devices are readily available for many abiotic sources of stress, such as temperature, humidity, CO_2_, and illumination. Furthermore, VOCs microsensors are becoming available in the market. However, these devices are not specific, and they provide only a generic evaluation of a total amount of volatile compounds. The information that these sensors can carry is very coarse and hardly usable for an investigation into the physiological changes occurring in the plant. However, the rate of production of VOCs is related to metabolic activity, and at least, this information can be gathered from these sensors.

Networks of wireless sensors have been proposed to monitor the conditions of plants in different conditions, for instance, in greenhouses [6]. In these applications, sensors were mostly aimed at detecting the environmental conditions [7]. The implementation of gas sensors in wireless sensor networks (WSNs) has been attempted for indoor air quality and pollution detection [8], forest fire detection [9], animal monitoring [10] and precision agriculture [11]. To our knowledge, no application of gas sensors in WSNs aimed at monitoring plant monitoring has been reported so far.

In this paper, we describe the development and application of a wireless sensor system aimed at detecting, in real-time, a number of physiological and environmental parameters. The sensor system has been designed with the scope of monitoring the conditions of small plants during their transportation. This is a niche application in the more general field of plant or crop monitoring. In this case, a limited quantity of plants, pot-planted and confined in a closed environment, are considered.

The considered quantities are illuminance, temperature, relative humidity, and total VOCs (TVOCs). The sensors have been assembled on the same board equipped with wireless data transmission. 

A variety of support is available for data transmission. Low-power wide-area networks (LPWANs) are particularly suitable to cover field crops, and even, to a certain extent, the transportation of plants. Different implementations of LPWANs are available, such as Sigfox, LoRa, and NB-IoT. Each protocol is characterized by different properties, but all of them are suitable for sensor network data exchange. Among them, LoRa ensures the coverage of a wider area and a low power consumption [12].

The transportation of plants can occur with a variety of vectors, including lorries, trains, or even ships. The LoRa protocol provides the basis for an “on-board” network which is not limited by either the size nor the physical conditions, such as barriers or containers.

As an example of application, the wireless sensor node (WSN) has been used to monitor the parameters of plants of the same species, size, and age, but kept in extremely different conditions. Both plants were planted in the same soil, but one plant was exposed to natural light and regularly watered, and the other was kept in the dark and without water.

Results showed that the TVOCs production is related to the health conditions. In particular, the plant kept in the dark and not watered produced a minor amount of volatile compounds. The relationship between environmental parameters and VOCs is evidenced by a neural network regression model that can estimate the amount of total VOCs from the environmental parameters.

## 2. Materials and Methods

### 2.1. Sensors Selection

Table 1 shows the sensors selected for the WSN. All these devices were chosen because of their low consumption and the digital output that allows for a simple and secure connection with the other devices on the WSN. 

The choice of integrated gas sensors on the market is rather limited. Here, the products of three different vendors were considered: Sensirion, Bosch, and AMS. Each of these devices is based on a metal–oxide semiconductor gas sensor technology [13]. They are sensor systems in which the gas sensor is integrated with read-out electronics, preprocessing, and digital output. As a negative characteristic, the developers of integrated gas sensors tend to seclude access to raw sensor data, and rather, they provide data elaborated by proprietary algorithms aimed at providing an evaluation of parameters such as the total VOCs (tVOCs). However, since sensors are rather unselective, these algorithms are optimized, keeping in mind particular applications.

For instance, the Bosch (BME680) [14] device provides as output an evaluation of the internal air quality (IAQ), obtained with internal preprocessing. This is a parameter conceived to evaluate the air quality of living spaces, and it is hardly usable for other contexts, such as the VOCs released by plants. In the device from Bosch, the evaluation of IAQ is based on the gas sensor and on three additional sensors of temperature, relative humidity, and pressure. The Sensirion device (SGP30) [15] integrates four metal–oxide semiconductor sensors, and the evaluation of total VOCs can be obtained with external processing in which gas sensor signals are integrated with the temperature value and the relative humidity (provided by SHT31) and applying a proprietary algorithm. The CCS811 sensor measures tVOCs as well, but, as tested, it is not fit for the application due to its vulnerability to high values of relative humidity [16].

All tested sensors, namely, SGP30, CCS811, and BME680, calculate an equivalent CO_2_. This parameter does not coincide with the real concentration of CO_2_ because it aims to provide an evaluation of CO_2_ produced by human breath. Such a value is likely to be not relevant in the case of plants. The most critical parameter for Metal Oxide (MOX) sensors is relative humidity, as already specified. CC811 may be seriously damaged at Relative Humidity (RH) above 95%, even for a short period. The response of BME680 to a low concentration of VOCs is saturated in the presence of a high RH, but this condition is reversible, and the sensor returns to work correctly as soon as RH decreases. Finally, SGP30 is the most robust to RH and, even if a combination of VOCs and RH may lead to saturation, it provides reliable signals in most of the cases. Ultimately, the SGP30 is the most suitable device for this application.

### 2.2. WSN Board Design

The WSN was designed for long range transmissions in order to be used to monitor crops and to follow the behavior of plants during their transportation. This last application is particularly useful for the ornamental plant market.

**Figure 1:**
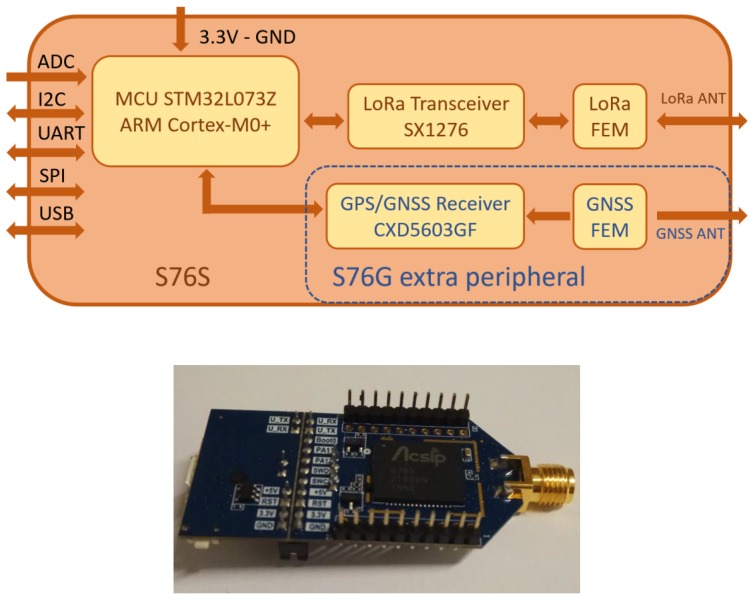
The simplified block diagram of the S76G chip, produced by Acsip, and the evaluation board of the S76G chip.

The design is centered on the S76G chip (Acsip Technology Corp., Taoyuan City, Taiwan). It integrates a STM32L0 ultralow-power MCU with an Arm^®^ Cortex^®^-M0+ core, a LoRa SX1276 Transceiver, and a Sony GNSS Receiver CXD5603GF for high precision position measurement. Figure 1 shows details of S76G. 

The microprocessor communicates with sensors via a shared I^2^C line, where all sensors are identified by a unique address code. The standard speed of transmission of 100 kbit/s suits all the sensors. It is the slowest available, but in this application speed, performance is not a priority.

Each sensor is mounted on a breakout board, with a linear voltage regulator, to adapt the alimentation voltage to the value required by the devices, and a voltage level shifter that permits the use of both the 3 V and 5 V communication logic.

In the initial state, the processor configures peripherals, such as timers and the LoRa module, then starts the initialization of sensors. In this process, it is possible to select for each device parameters of measurement, such as sample rate and others. Most of the devices permit a choice between different operating modes, which differ in precision, time duration of the detection, and power consumption. Where possible, maximum accuracy is always selected. After the initialization, the microprocessor starts to communicate periodically with sensors and to collect data, storing them into arrays, that will be later used to create the buffer to send to the master via LoRa communication.

The system is implemented in both a normal and low power mode, where the microprocessor periodically enters into stop mode, to exploit the power consumption reduction. Differences between these two implementations are explained later.

The communication of data is based on the star network architecture, where one of the WSNs acts as a master. Thus, it collects data from the other WSNs, the slaves, and then communicates them to a PC via UART protocol. 

### 2.3. WSN Test on Plants

WSNs were tested in two experiments. In each experiment, two plants cultivated in the same soil and contained in similar plant pots were kept for 11 days. Measurements were performed on European privet (*Ligustrum vulgare* L., 1753).

Plants were kept indoors. One was exposed to natural light and watered daily, while the other was not watered and kept in the dark. Plants were kept in a plastic container sealed enough to allow for the measurement of plant-released VOCs but with a sufficient air inlet.

**Figure 2:**
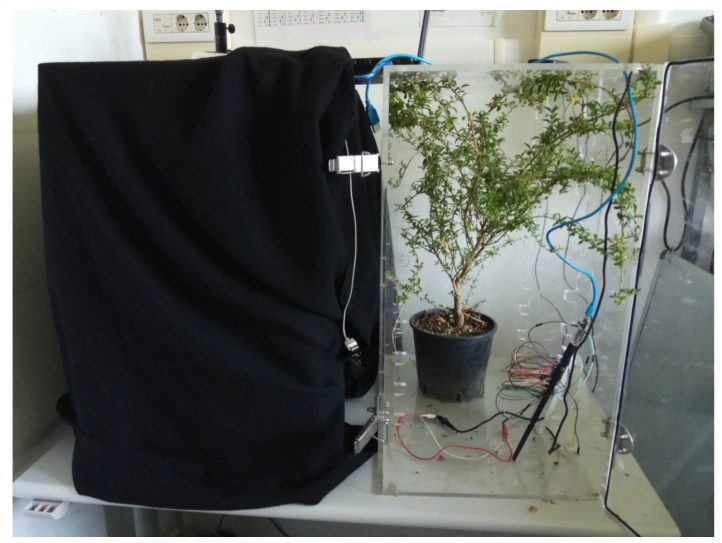
WSN test of plants enclosed in transparent boxes in order to simulate transport inside lorries. The transparent box shows an enclosed European privet. WSN and its antenna are also visible inside the box. The box is endowed with a series of holes for air circulation.

Figure 2 shows a picture of the two plant containers. WSNs were continually connected, via LoRa, to a remote computer, and data were uploaded every 5 min. At this rate, a good compromise between monitoring precision, power consumption, and data storage is achieved. Indeed, a higher rate of sampling would only increase the power consumption of the node without a relevant difference in the information collection.

## 3. Results and Discussion

### 3.1. Functional Tests

The sensor node is composed of two different parts: the S76S/S76G board and the array of sensors. Both are powered by the same 3.6 V LiPo battery. To analyze appropriately the power consumption of the entire node, measurements were performed on both parts. In Table 2, the values measured are reported, not only for the node, which is the slave in the network, but also for the master. The central node, of course, requires more energy, because it is constantly in receiving mode, waiting for messages from slaves. Data for the slave board are reported in both normal mode (NM) and low-power mode (LPM), for comparison. The integration of the stop mode permits a good reduction in the power dissipation of the node. Array (a) of sensors is composed of all sensors previously described, except the CCS811. In array (b), the SGP30 was also removed to highlight that most of the power consumption comes from this device. Through adding together the consumption of the board and of the array, the total power dissipation of the node is obtained.

Multiple tests were performed to understand the range of the network in different urban conditions. Distances are considered valid if most of the packets, 98–99%, are received correctly by the master, because missed receptions correspond to data loss and the usage of acknowledgements that would increase the consumption of nodes. In the communication implemented, due to the absence of acknowledgements, missed receptions correspond to data loss, then distances are considered valid if most of the packets are received correctly by the master, for example, 98–99%. To further increase the area covered by the communication, it is possible to implement retransmissions, even if this has a negative impact on the power consumption of the node. 

### 3.2. Plant Experiment

WSNs were used to monitor the parameters of two European privets (*Ligustrum vulgare* L., 1753) kept in radically different conditions. One plant was kept in the dark without watering while the other was exposed to natural light and watered daily. Measurements were continuously acquired for 11 days.

**Figure 3:**
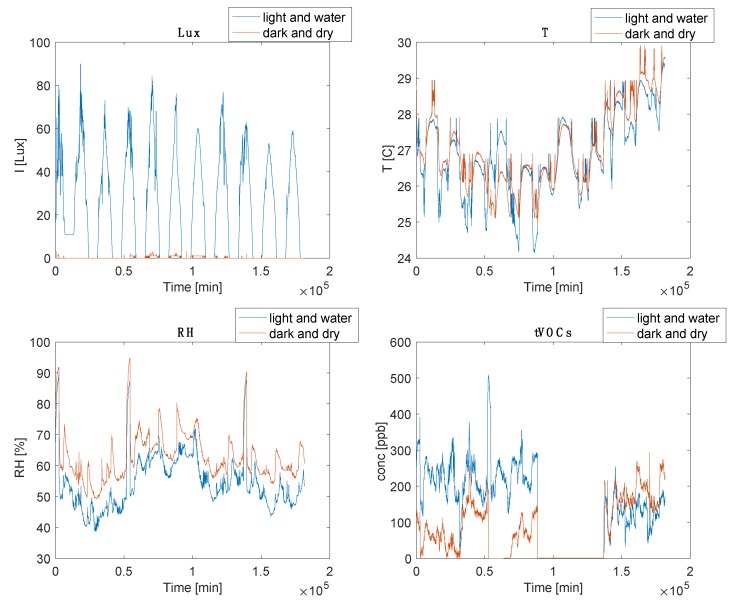
Behavior of the signals provided by the four sensors. Gas sensors experienced short failures.

Figure 3 shows the data provided by the four sensors. Ambient data were regularly recorded while the gas sensor experienced two malfunction periods. Oscillations in temperature and humidity follow the environmental conditions. However it is interesting to note that the humidity in the box kept in the dark was higher than that detected in the container exposed to natural light, and this might be related to the stress condition of the plant. The behavior of the total VOCs is rather erratic and correlated poorly with the other variables. The gas sensor experienced two periods of malfunctioning due to a fast increase of RH in the measurement chamber. These periods have been removed from the following analysis.

A better understanding of the relationship between the four parameters can be acquired through a multicomponent analysis. For the scope, the data were processed with the principal component analysis (PCA) [17]. Calculations were performed with the statistics toolbox of MATLAB.

**Figure 4:**
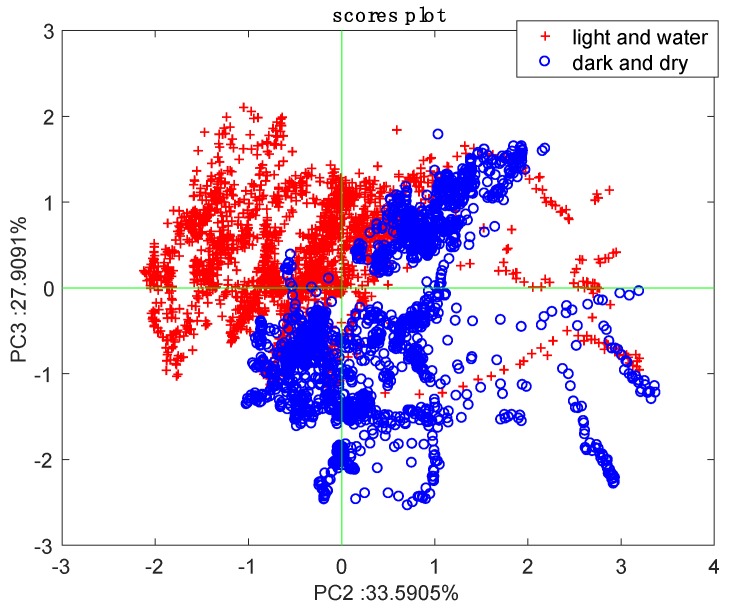
Principal component analysis (PCA) scores plot based on temperature, relative humidity, and total VOCs measured across 11 days.

Since the illumination is drastically different, it was eliminated from the calculus of PCA. The first principal component carries most of the quantitative information; thus, the separation between the two plants is rather obvious and it can be directly observed in Figure 3. More interesting is to observe the behavior of the other two principal components where the influence of the magnitude of signals is reduced. The results of PCA are manifested by the scores and loadings plots. Figure 4 shows the scores plot with a clear separation between the two conditions. It is worth considering that the measures are dynamically evolving, and each parameter spans an overlap range of values throughout the day. Thus, the fact that most of the time, the two conditions are clearly separated is a not trivial result.

**Figure 5:**
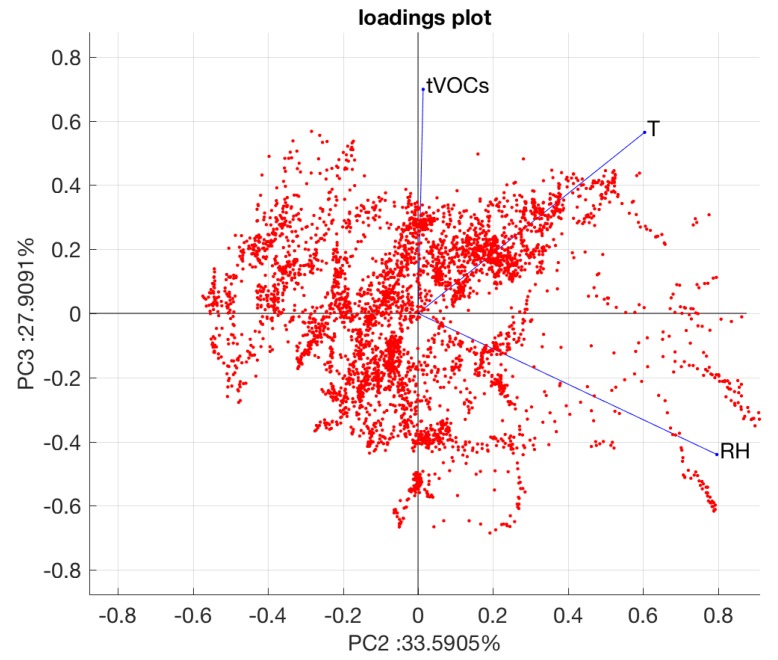
Biplot of the PCA.

The loadings provide information about the role of each parameter to the scores plot. The loadings are optimally appreciated in a biplot where the directions of the original axis are projected onto the scores plot. The biplot of the data is shown in Figure 5.

The three variables are divergent from each other, which shows that each variable contributes independently to the scores plot. This is particular evident for relative humidity and total VOCs. The separation between the two conditions is minimally influenced by the temperature, but it is rather dependent on total VOCs and humidity.

PCA evidence shows the interrelation between the environmental parameters and the released VOCs. Thus, it is usual to study the possibility to retrieve the amount of total VOCs from the environmental parameters. It is worth remarking that other than the environmental conditions, the plants are absolutely identical and share the same initial conditions.

In this regard, it is also important to consider that each data point is not independent from those following and preceding; thus, any regression method must consider the time evolution of the ambience and of the plant status. The optimal way to study such a system is to consider the data as a time series. After the elimination of the data corresponding to the malfunction of the gas sensor, the experiment collected 2225 data from the illuminated plant and 1952 data from the plant kept in the dark. Two thirds of each group has been taken for training and the final third for testing the model.

The regression has been carried out with a recursive neural network implementing a nonlinear autoregressive model with external inputs (NARX) [18]. In practice, the series of total VOCs was predicted given a number of past data of total VOCs and environmental parameters. The neural network model was calculated using the Neural Network toolbox of Matlab. A total of 10% of training data were used for internal validation and another 10% for internal testing. The network was made of 10 hidden neurons and 3 inputs (illumination, temperature, and relative humidity) and 1 target output (total VOCS). The delay was fixed to 2. This means that to estimate the amount of VOCs at the time t, the network uses the environmental parameters at time t, t-1, and t-2, and the estimated amount of VOCs at times t-1 and t-2. Considering that data are taken every 5 min, the estimation of VOCs at time t is based on the measurement and estimation back to 15 min.

**Figure 6:**
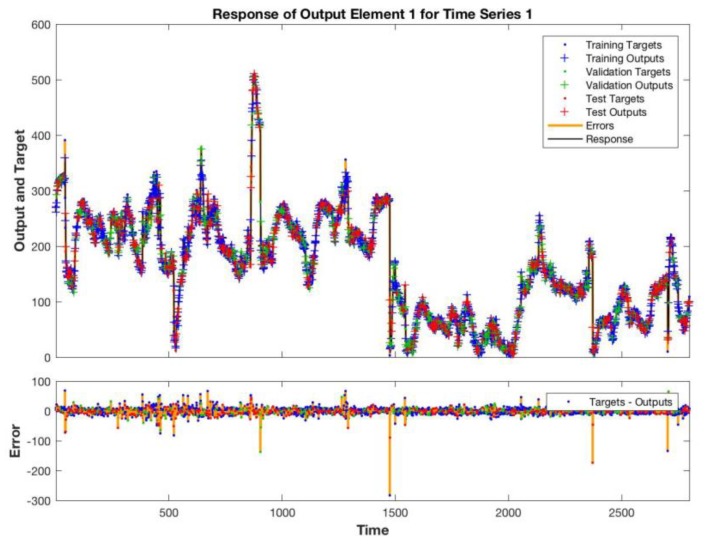
Estimated and measured total VOCs. The bottom plot shows the error between estimation and measured values.

**Figure 7:**
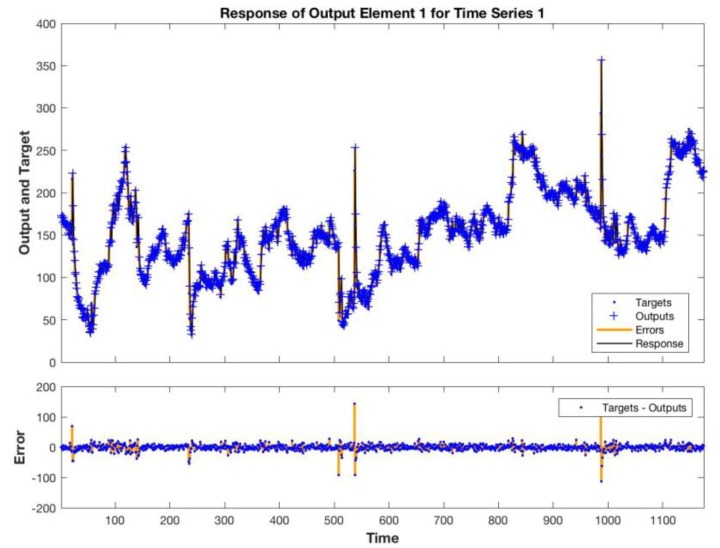
Estimated and measured total VOCs.

**Figure 8:**
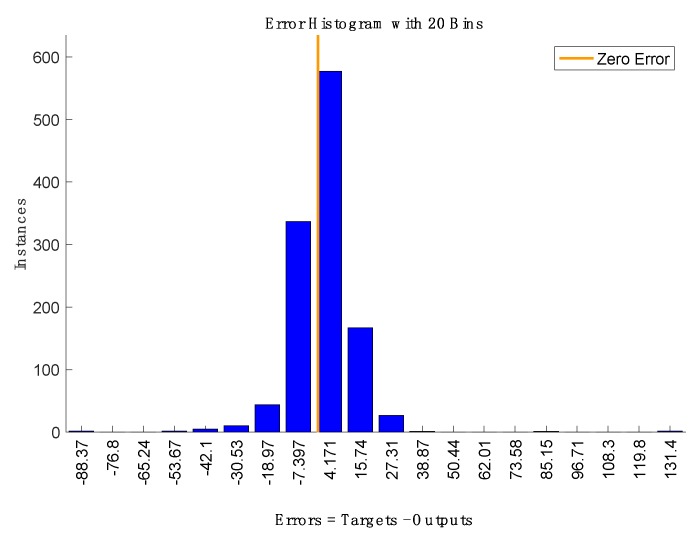
Error histogram during test.

Figure 6 shows the sequence of estimated and measured total VOCs in the training data set. Large errors are achieved at the transitions between sequences of data. In correspondence of these points, the temporal relationship between data is obviously lost. Figure 7 shows the network estimate of the test data set, which corresponds to the last sequence of data of both plants. In Figure 8, the histogram of the errors is shown. Most of the errors are in the interval between −7 and + 15 ppb, only 3% of the whole range of variability of the total VOCs.

## 4. Conclusions

In this paper, a network of WSNs for monitoring environmental parameters and the total amount of VOCs released by a plant has been illustrated. The technology of ambient sensors (illumination, temperature, and relative humidity) is rather solid, and a number of sensors of good performance, limited cost, and low-power consumption are available. However, the recently introduced integrated gas sensors in CMOS technology are complemented by an internal processing mainly aimed at compensating typical drawbacks of these sensors, such as drift and lack of selectivity. However, the processing is in almost all cases addressed towards the evaluation of qualitative parameters of indoor air quality. This is a quantity of almost null interest in plant monitoring.

Here, we adopted a sensor which provides an evaluation of total VOCs. Interestingly, we found that the total VOCs are discriminative of the different conditions in which the plants are kept. Hence, even if this quantity is only a coarse evaluation of the real air composition, it was shown to be useful to monitor stress events in plants. It is important to emphasize that for a thorough interpretation of the sensors’ data, a deep investigation of the physiology of the plants under stress is necessary. Furthermore, the final objective of the WSN illustrated here is the detection of stress in potted plants and during their transportation; thus, the conditions of such plants may be quite different from that of those cultivated in natural soil and exposed to atmospheric agents.

As a further demonstration, a recursive neural network model shows that the total VOCs can be derived from the environmental parameters. This is not surprising, considering that the plants were absolutely identical and planted in the same soil, and the only differences between them were the environmental conditions. This result cannot be straightforwardly extrapolated to all plants in all conditions, but it provides a solid basis for the monitoring of a crop where all plants follow a parallel development and are exposed to the same treatment and environmental situations.

During transportation, plants are likely to be kept together in a closed environment where they share the same physical conditions. In this situation, the measure of VOCs from single plants is likely influenced by the VOCs released by the other plants and the extent of this influence depends on the distribution of heating, ventilation, and air conditioning (HVAC) inside the container. In these conditions, the deployment of WSN combined with a proper data analysis, integrated with HVAC system parameters, could be used to optimize the HVAC system to ensure homogeneous conditions. 

## Figures and Tables

**Table 1:** Sensors implemented in the wireless sensor networks (WSNs).

Quantity	Device	Vendor	Dimension	Consumption
Illuminance [lux]	TSL2561	Adafruit	3.80 × 2.60 mm^2^	750 µW
Temperature [°C] and relative humidity [%RH]	SHT31	Sensirion	2.5 × 2.5 mm^2^	5 µW
Total VOCs [ppb]	SGP30	Sensirion	2.45 × 2.45 mm^2^	150 mW
Total VOCs [ppb]	CCS811	AMS	2.7 × 4.0 mm^2^	46 mW
Air quality index	BME680	Bosch	3.0 × 3.0 mm^2^	36 mW

**Table 2:** This table summarizes the power consumption statistics for each device of the system. S76S NM stands for normal mode. S76S LPM stands for low power mode. Array (a) is the array of sensors described without the CCS811 device. Array (b) is the array of sensors without CCS811 and SGP30.

Power Consumption	Master	S76S NM	S76S LPM	Array (a)	Array (b)
**Energy [mWh]**	180 mWh	50.4 mWh	28.8 mWh	158.4 mWh	6.6 mWh
**Electric Charge [mAh]**	36 mAh	14 mAh	8 mAh	48 mAh	2 mAh
